# Yield, Growth, Quality, Biochemical Characteristics and Elemental Composition of Plant Parts of Celery Leafy, Stalk and Root Types Grown in the Northern Hemisphere

**DOI:** 10.3390/plants9040484

**Published:** 2020-04-09

**Authors:** Nadezhda A. Golubkina, Viktor A. Kharchenko, Anastasia I. Moldovan, Andrey A. Koshevarov, Svetlana Zamana, Sergey Nadezhkin, Alexey Soldatenko, Agnieszka Sekara, Alessio Tallarita, Gianluca Caruso

**Affiliations:** 1 Federal Scientific Center of Vegetable Production, Selectsionnaya 14, Vniissok, Odintsovo district, Moscow region 143072, Russia; kharchenkoviktor777@gmail.com (V.A.K.); nastiamoldovan@mail.ru (A.I.M.); zato@inbox.ru (A.A.K.); nadegs@yandex.ru (S.N.); alex-soldat@mail.ru (A.S.); 2 State University of Land Management, Kazakova str. 15, 10506 Moscow, Russia; svetlana.zamana@gmail.com; 3 Department of Horticulture, Faculty of Biotechnology and Horticulture, University of Agriculture, 31-120 Krakow, Poland; agnieszka.sekara@urk.edu.pl; 4 Department of Agricultural Sciences, University of Naples Federico II, 80055 Portici (Naples), Italy; lexvincentall@gmail.com (A.T.); gcaruso@unina.it (G.C.)

**Keywords:** *Apium graveolens* L., plant parts, sugars, polyphenols, flavonoids, carotenes, chlorophyll, antioxidant activity

## Abstract

Celery is one of the major nutraceutical vegetable species due to the high dietary and medicinal properties of all of its plant parts. Yield, growth and produce quality of six celery genotypes belonging to leafy (Elixir and Samurai), stalk (Atlant and Primus) or root (Egor and Dobrynya) types, as well as the distribution of biomass, sugars, mineral elements and antioxidants among the different plant parts, were assessed. Within the celery root type, cultivar Dobrynya resulted in higher yield than Egor, whereas the genotype did not significantly affect the marketable plant part production of leafy and stalk types. Leaf/petiole ratios relevant to biomass, total dissolved solids, sugars, ascorbic acid, flavonoids, antioxidant activity and ash, K, Zn, Fe, Mn, Cu and Se content were significantly affected by the celery type examined. Ash content was highest in the leaves and lowest in the roots. Celery antioxidant system was characterized by highly significant relationships between ascorbic acid, polyphenols, flavonoids, antioxidant activity and Zn. Among the celery types analyzed, the highest values of chlorophyll, Fe and Mn content as well as antioxidant activity were recorded in leaves from root genotypes, which suggests interesting nutraceutical prospects of the aforementioned plant parts for human utilization.

## 1. Introduction

Celery (*Apium graveolens* L.) is a vegetable with a high nutraceutical value, that is, an excellent source of antioxidants and minerals beneficial to human health, and is widely used in traditional medicine and ethnoscience [1]. In particular, this plant species is characterized by high contents of ellagic, protocatechuic, chlorogenic and gallic acids [2], quercetin, kaempferol, apigenin and luteolin [1], and a high percentage of essential oils; the latter is mainly represented by phthalides, known for their anti-inflammatory, antitumor and insecticidal properties [3,4,5]. The wide biological activity of celery gives the opportunity to prevent and treat several diseases, such as cardiovascular [6], liver and spleen diseases [7], rheumatism [8], inflammation [9], cancer [10] and gastrointestinal disorders [11]. Studies carried out on laboratory animals showed that the ethanolic extract of celery leaves increased spermatogenesis [12] and improved fertility [13,14]. In addition, celery was found to decrease glucose level and improve lipid profile in blood [15], and stabilize blood pressure [16].

All parts of celery plants are edible: leaves, petioles, roots and seeds, either fresh or canned, or dry as spices [17]. In this respect, despite the wide utilization of *Apium graveolens* L., up-to-date research has not paid much attention to the comparative assessment of biochemical characteristics of different celery types, namely, leafy, stalk and root genotypes [2]. The investigation of Sellami et al. [3] demonstrated the peculiarities of essential oil composition of celery leaves, stalks and roots, but did not compare different celery types.

Gaining a more in-depth knowledge regarding the aforementioned topics may lead to unveiling the nutrient and antioxidant accumulation potential in celery plants, which is of great importance for targeting functional food production.

The present investigation was aimed to assess yield, growth and produce quality of leafy, stalk and root celery types, as well as the distribution of biomass, organic and mineral compounds, and antioxidants among the different plant parts, grown in the Northern Hemisphere. The results were frequently expressed as ratios between the variables assessed, which showed lower variability than the absolute values.

## 2. Results and Discussion

### 2.1. Yield, Morphobiological Parameters, Dry Matter, Total Dissolved Solids and Nitrate Content

The data presented in Table 1 suggested that, among the celery morphobiological characteristics examined, the stalk/leaf biomass ratio varied from 1 in root celery to 1.5 in leafy and 2.6 in stalk types. Indeed, stalk is a storage plant part in leafy and petiole celery genotypes, playing a minor storage role in root celery, which is consistent with the results obtained in the present study. Notably, similar biomass values of leaves and petioles in root celery may reflect the process of intensive nutrients’ transport from leaves to roots, whereas the high petiole/leaf biomass ratio in stalk celery can be considered as a favorable trait for plant productivity.

Leaf surface was similar in leafy and root types, despite significant morphological differences in leaves’ structure, and was the smallest in celery stalk types (Table 1). Leaf surface is one of the structural–functional parameters of the photosynthetic apparatus and is directly related to plant productivity. The leafy celery cultivar Elixir demonstrated both higher leaf surface and plant biomass compared to cultivar Samurai. A similar phenomenon was recorded in root celery cultivars: higher root biomass was shown by cultivar Dobrynya, whose leaf surface was 1.35 times wider than that of cultivar Egor.

Fewer variations were recorded for leaf/petiole dry matter content and were in the range of 1.33–1.42 in all celery types (Table 2). The dry matter content in celery roots was closer to that detected in leaves. Despite the predominant accumulation of water in petioles, the distribution of dissolved compounds (TDS, total dissolved solids) in water between leaves and petioles greatly differed between leafy, stalk and root celery types. The TDS content in leaves was twice higher than that in petioles, both in leafy and stalk celery types. In contrast, root celery showed the same amount of TDS both in leaves and petioles (Table 2), suggesting carbohydrate transfer from leaves to roots in root celery.

Among the agricultural crops, celery belongs to a group of plants characterized by a genetically determined high concentration of nitrates [18], which is confirmed by the results of this research. The highest nitrate levels were recorded in the root type cultivar Dobrynya, which showed similar nitrate levels in leaves and petioles. In contrast, nitrate concentration was higher in leaves in the leafy and stalk types (Table 2). Furthermore, leaf/petiole nitrate ratio in leafy and stalk celery types were in the range of 2.00–2.15, while this parameter in root celery was much lower (i.e., from 1.0 to 1.47). This data relevant to the high nitrate accumulation in leaves and petioles of root celery should not be considered as a risk factor of carcinogenic N-nitrosamine formation in humans [19], due to low consumption levels and relatively high concentrations of antioxidants preventing the synthesis of N-nitrosamines [18].

Mannitol, glucose and fructose are the major monosaccharides in celery [20], while sucrose is the main sugar storage form in celery roots. Petioles are the storage parts in leafy, stalk and to a lesser extent in root celery plants. Therefore, petioles accumulated higher amounts of total sugar compared to leaves, with predominance of monosaccharides (Table 2).

The mono-/di-saccharide ratio was of special interest. The ratio was in the range of 1.3–3.9 in leafy and root types, whereas it reached 6.7–6.8 in the stalk types. The latter phenomenon clearly reflects the sweeter taste of petioles in stalk celery compared to leafy and root types. Interestingly, the ratio of monosaccharide accumulation between petioles and leaves tended to the value 2 in cultivars Elixir, Atlant and Dobrynya or to the value 1.61 in Samurai and Egor.

### 2.2. Antioxidants and Photosynthetic Pigments

All the investigated celery types showed high levels of ascorbic acid in leaves (Table 3). Notably, compared to petioles, the leaf ascorbic acid concentration was 5 to 6 times higher in the case of leafy and stalk types, and 8 times higher in root genotypes where the content of this antioxidant in petioles was not significantly different from that recorded in roots. Ascorbic acid is a widespread plant metabolite that is present in all subcellular structures and is essential for plant functioning. It is an important antioxidant and enzyme cofactor, participating in photosynthesis regulation, hormone biosynthesis and regulation of activity of other antioxidants. It regulates cell division, plant growth and development, including flowering, senescence, root evolution, and supports plant defenses under biotic and abiotic stress conditions [21,22].

The content of phenolics was higher in leaves than in petioles in leafy and stalk types, and was the highest in leaves in root genotypes. Higher antioxidant activity and lower flavonoid content were recorded in leaves of root celery compared to leaves of the other celery types (Table 3).

Overall, the celery antioxidant system was characterized by similar, high levels of ascorbic acid in leaves of leafy, stalk and root celery types; significant prevalence of antioxidants (ascorbic acid, polyphenols and flavonoids) in leaves compared to petioles; and higher flavonoid levels in leaves of stalk and leafy types compared to root celery. Salehi et al. [1] reports that celery flavonoids are represented by miricetin, quercetin, kaempferol, luteolin and apigenin, and the high levels of antioxidants determine its resistance to oxidative stress. Indeed, quercetin, kaempferol and apigenin exert strong anticarcinogenic effects by promoting apoptosis [23], and luteolin, quercetin and kaempferol display inhibition of estrogenic action [24].

A significant positive correlation between antioxidants and dry matter content was recorded in this study (Table 4), along with positive correlations between all the investigated antioxidants (ascorbic acid, polyphenols, flavonoids and antioxidant activity), indicating a close relationship between the different components of the celery antioxidant system.

A predominant accumulation of monosaccharides in petioles containing lower levels of antioxidants explained the negative correlation between these two parameters (Table 4). Notably, a highly significant positive correlation was recorded between ash content and total antioxidant activity, which may suggest the participation of mineral elements in the plant antioxidant system.

**Figure 1:**
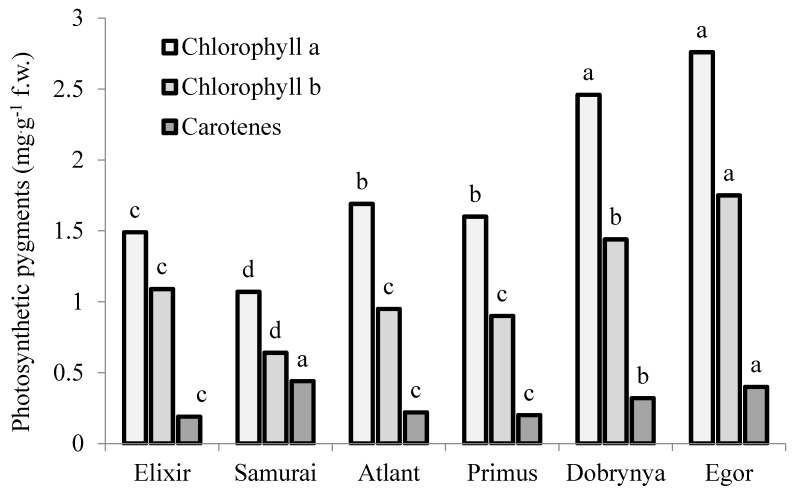
Photosynthetic pigment content in different plant parts of celery cultivars: leaves (Elixir and Samurai), petioles (Atlant and Primus) and roots (Dobrynya and Egor). Values followed by different letters are significantly different according to Duncan’s test at *p* ≤ 0.05.

The leaves of root celery types were characterized by the highest levels of chlorophyll a and b, giving the opportunity to plants to synthesize sugars more intensively, which is closely connected with sugar accumulation in roots (Figure 1). Despite the difference in chlorophyll content between the three celery types studied, chlorophyll a/chlorophyll b ratio remained constant at 1.6–1.7.

### 2.3. Content of K, Zn, Mn, Fe, Cu and Se in Plant Parts

The investigated macro elements and microelements are involved in the plant antioxidant system [25,26,27] and, in this respect, the mineral composition in celery reflects the degree of nutrient supply, tolerance to biotic and abiotic stressors and nutritional value of produce. The data presented in Table 5 show the significant differences in mineral content recorded between the different plant parts and between cultivars, as well as the distribution of the elements analyzed among leaves, petioles and roots. The highest ash content was recorded in cultivar Dobrynya and the lowest ash content was recorded in the leafy celery cultivar Elixir. In the Moscow region, under the application of ammonium sulfate, ammonium nitrate, superphosphate and potassium sulphate, the ash content in celery plants decreased from leaves to petioles and roots. Notably, according to literature reports, macro element distribution between leaves and petioles in celery may significantly vary depending on the cultivar and fertilizer applied [28]. In the present study, higher ash level in celery leaves compared to petioles was not connected with the plant’s ability to accumulate potassium, as an opposite leaf/petiole distribution was recorded for potassium. In this respect, the high ash content in leaves may be correlated with the content of Ca and Mg, which predominantly accumulate in leaves [29].

Other investigations reported the significant influence of P, Ca and Mg supply on celery leaf elemental composition [30].

Potassium is known to reduce blood pressure, which suggests the high medicinal value of celery petioles as a major source of potassium. Indeed, 200 g of petioles from leafy and stalk celery types provide about 1.17–1.44 mg of K, which corresponds to 59%–72% of the adequate consumption level (2 g per day). Leaves and petioles of root celery genotypes accumulate 2 to 3 times higher amounts of Fe than leafy and stalk types. These values correspond to 2–3 mg Fe in 50 g of fresh leaves of root celery, that is, 11%–17% of the adequate consumption level for Fe (18 mg RDA). Though plant Fe is less available for human organism than meat iron, the presence of antioxidants in vegetables (in particular, ascorbic acid) is known to provide a 3-fold increase in Fe bioavailability [31]. The results of the present study demonstrated the higher antioxidant activity of root celery leaves compared to leafy and stalk types and, accordingly, indicated the prospective use of root celery leaves in human nutrition.

The metabolism of iron and manganese is closely connected to each other in plants [32]. The analysis of celery elemental composition revealed that root celery leaves contained a twice higher concentration of Mn compared to leafy and stalk types. In particular, 50 g of fresh root celery leaves provide about 0.3 mg of Mn, that is, about 13% of the adequate consumption level of this element. Being a part of vitally important enzymes, Mn improves vitamins E and B_1_ absorption, optimizes nervous system activity, prevents depression, improves memory and thyroid function and is considered to be a key factor in glucose uptake by brain and in neurotransmitter control. In this respect, leaves of root celery could be of great importance in human nutrition, though they have been rarely used for this purpose so far.

In the present research, zinc distribution between petioles and leaves in different celery types indicated significantly higher levels in leaves with Zn leaf/petiole ratio of 1.22–1.28 in leafy and stalk types and 2.46–2.36 in root celery. The latter phenomenon can be explained by the involvement of zinc in building up chlorophyll and carbohydrates, which ensures the resistance of celery plants to cold stress. Lower Zn levels in root celery petioles compared to leafy and stalk types may be connected with the important role of Zn in root formation, including auxin synthesis, cell membrane integrity and ion transport [33].

Though Zn does not participate in redox reactions, its function as an antioxidant is to catalyze the activity of Cu/Zn superoxide dismutase, stabilize the structure of membranes, protect sulfhydryl groups in proteins and regulate the expression of metallothioneins with metal binding and antioxidant ability. The correlation analysis demonstrated the participation of Zn in plant antioxidant system (Table 4). Moreover, the relationships between different antioxidants in celery plants may be described by the following scheme:


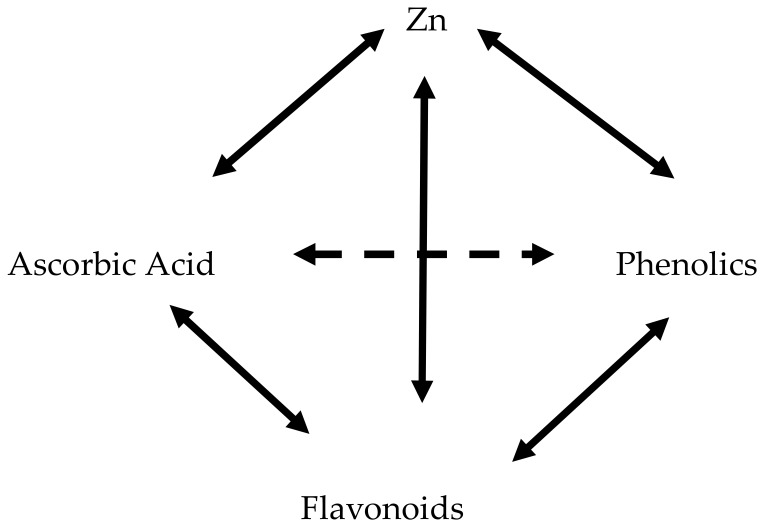


The peculiarities of the celery antioxidant system, reflected in the strong relationships between ascorbic acid, flavonoids, phenolics, antioxidant activity and Zn, were shown in celery in the present study. In a previous research [34], highly significant relationships between ascorbic acid, phenolics, antioxidant activity, Se and K, but not Zn, were recorded in leek. The latter phenomenon may be explained by the fact that, unlike celery, leek belongs to secondary accumulators of selenium. The data in Table 4 suggest that, despite the possible Se participation in plant antioxidant defense [27], selenium did not show any relationships with the celery antioxidant system components.

**Figure 2:**
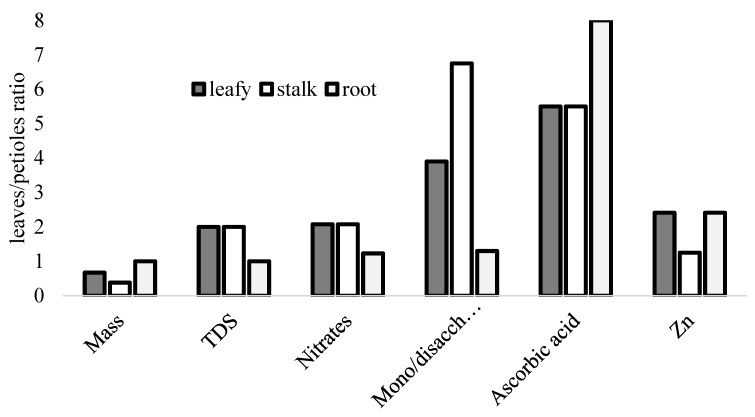
Typical leaf/petiole ratios in leafy, stalk and root celery types.

Typical ratios of leaf/petiole parameters are presented in Figure 2.

## 3. Material and Methods

### 3.1. Growing Conditions and Experimental Protocol

The research was carried out in 2018 and 2019 on celery (*Apium graveolens* L.), grown at the experimental fields of the Federal Scientific Center of Vegetable Production (Moscow region, 55°39.51’ N, 37°12.23’ E), on sod-podzolic clay loam soil (pH 6.8, 2.1% organic matter, 1.1 g·kg^−1^ N, 0.045 g·kg^−1^ P_2_O_5_, 0.357 g·kg^−1^ K_2_O). The mean values of temperature (°C) and relative humidity (%) as an average of 2018 and 2019 were the following: 16.1 °C and 71.8% in May, 21.0 °C and 73.0% in June, 23.8 °C and 74.9% in July, 19.0 °C and 76.9% in August and 14.8 °C and 86.0% in September, respectively.

The effects of three celery types (leafy, stalk and root), with each type including two cultivars (leafy type, Elixir and Samurai; root type, Egor and Dobrynya; stalk type, Atlant and Primus) were assessed on leaves, petioles and roots in terms of yield, biomass, quality attributes, antioxidants and elemental composition. The treatments were distributed in the field according to a randomized complete block design with three replicates and each experimental unit had a surface area of 10.5 m^2^ (3.5 × 3.0 m).

Seedlings with three true leaves were transplanted in an open field on 21 and 22 May in 2018 and 2019, spaced 35 cm along the rows, which were 60 cm apart. Fertilization was practiced by supplying 30 kg·ha^−1^ N (ammonium sulfate), 60 kg·ha^−1^ P_2_O_5_ (superphosphate) and 100 kg·ha^−1^ (potassium sulfate) prior to planting, and 50 kg·ha^−1^ N (ammonium nitrate) during the crop cycle in two applications, two and five weeks after transplant, respectively. Irrigation was practiced by a sprinkler system when the soil available water dropped to 65%–75%; and soil available water was evaluated on random samples, taken every two days from 0 to 30 cm depth and transferred to a laboratory, by using the gravimetric method.

At harvest, carried out at the beginning of October, the following determinations were performed in all plots: weight of edible parts in each celery type and biomass as well as leaf number and area per plant.

### 3.2. Sample Preparation

After harvesting leaves, stalks and roots were separated and weighed, roots were washed with water and dried with a filter paper. Samples were homogenized and fresh homogenates were used for the determination of ascorbic acid, nitrates and water-soluble compound (TDS) content. A portion of the samples was dried at 70 °C to constant weight and was used for the determination of polyphenols, flavonoid content, total antioxidant activity and mineral composition.

### 3.3. Dry Matter

The dry residue was assessed gravimetrically by drying the samples in an oven at 70 °C until constant weight. 

### 3.4. Ascorbic Acid

The ascorbic acid content was determined by visual titration of plant extracts in 6% trichloroacetic acid with Tillman’s reagent [35]. Three grams of fresh leaves/petioles/roots of celery was homogenized in a porcelain mortar with 5 mL of 6% trichloroacetic acid and quantitatively transferred to a measuring cylinder. The volume was brought to 60 mL using trichloroacetic acid and 15 min later, the mixture was filtered through a filter paper. The concentration of ascorbic acid was determined from the amount of Tillman’s reagent that went into titration of the sample. 

### 3.5. Preparation of Ethanolic Extracts

One gram of dry leaves or petioles or roots powder was extracted with 20 mL of 70% ethanol at 80 °C over 1 h. The mixture was cooled and quantitatively transferred to a volumetric flask, and the volume was adjusted to 25 mL. The mixture was filtered through a filter paper and used for the determination of polyphenols, flavonoids and total antioxidant activity.

### 3.6. Polyphenols

Polyphenols were determined spectrophotometrically using the Folin–Ciocalteu colorimetric method according to [36]. The concentration of polyphenols was calculated according to the absorption of the reaction mixture at 730 nm using 0.02% gallic acid as an external standard.

### 3.7. Antioxidant Activity (AOA) 

The antioxidant activity was evaluated via titration of 0.01 N KMnO_4_ solution with ethanolic extracts of dry samples [37].

### 3.8. Flavonoids

The total flavonoid content was determined by a spectrophotometric method based on flavonoid–aluminum chloride (AlCl_3_) complexation [38]. In this method, 0.5 mL of the ethanolic extract was diluted with 1.5 mL of 70% ethanol; and 0.1 mL of 2% AlCl_3_, 0.5 mL of 1 M sodium acetate solution and 1 mL of distilled water were added. The mixture was left for 30 min at room temperature and the absorption at 415 nm was measured. The total flavonoid content was determined using quercetin (Fluka, Switzerland) as an external standard.

### 3.9. Total Dissolved Solids (TDS)

TDS was determined in water extracts using a TDS-3 conductometer (HM Digital, Inc., Seoul, Korea).

### 3.10. Nitrates

Nitrates were assessed using a ion-selective electrode on ionomer Expert-001 (Econix, Russia), according to [39].

### 3.11. Selenium

The fluorimetric method was used for the determination of selenium content [40]. The precision of the results was verified by using a reference standard (lyophilized cabbage with Se concentration of 150 μg·kg^−1^) in each determination.

### 3.12. Elemental Composition

The content of Fe, Mn, Cu and Zn in leaves, petioles and roots of celery was determined using the Atomic Absorption Spectroscopy (spectrophotometer Shumatzu-7000) after acidic digestion of samples.

### 3.13. Photosynthetic Pigments

Chlorophyll a, chlorophyll b and carotene contents in leaves were assessed using a 98% ethanolic extract and reading its absorbance at 470, 649 and 664 nm according to the method described by Lichtenthaler [41].

### 3.14. Statistical Analysis

Data were processed by analysis of variance and mean separations were performed through Duncan’s multiple range test, with reference to 0.05 probability level, using SPSS software version 21. Data expressed as percentages were subjected to angular transformation before processing.

## 4. Conclusions

The comparative assessment of three celery types (leafy, stalk and root) revealed the differences in biomass accumulation, quality, elemental composition, antioxidant compounds and activity between leaves, petioles and roots. In addition to the interest in leaf, stalk or root production depending on the celery type, significant positive correlations between antioxidants and trace elements were also observed. In particular, the high content of biologically active compounds, Fe and Mn, recorded in the leaves from root celery genotypes suggests that the latter plant part is a high promising source of beneficial substances for human nutraceutical supply.

## Figures and Tables

**Table 1:** Yield and growth parameters of leafy, stalk and root celery types.

	Celery Type					
Parameter	Leafy		Stalk		Root	
	Elixir	Samurai	Atlant	Primus	Egor	Dobrynya
Plant total biomass (g)	1202 ± 84a	932 ± 72b	1001 ± 65b	953 ± 68b	707 ± 68c	930 ± 93b
*Yield (t·ha^-1^)	40.0 ± 3.9a	38.0 ± 2.7a	33.0 ± 2.0b	31.9 ± 1.5b	30.5 ± 0.9b	38.1 ± 0.8a
Mean root weight (g)	215 ± 21c	167 ± 17c	196 ± 20c	190 ± 23c	510 ± 51b	644 ± 64a
No. of leaves per plant	154 ± 11a	84 ± 7b	42 ± 4c	40 ± 3c	11 ± 4d	14 ± 6d
Leaf area (cm^2^)	377.9 ± 26a	284.1 ± 22b	192.9 ± 16c	188 ± 18c	272.1 ± 23b	366.6 ± 27a

The values followed by different letters are significantly different according to Duncan’s test at *p* ≤ 0.05. *Yield comparisons were performed along each line and within each celery type.

**Table 2:** Quality parameters and nitrate concentration as affected by celery type and plant part.

		Celery Type					
Parameter	Plant Part	Leafy		Stalk		Root	
		Elixir	Samurai	Atlant	Primus	Egor	Dobrynya
Dry matter (g·100 g^-1^ f.w.)	Leaves	20.6 ± 1.6aA	21.3 ± 1.6aA	21.0 ± 1.6aA	20.6 ± 1.4aA	21.2 ± 1.5aA	17.5 ± 1.3bA
	Petioles	15.5 ± 1.2aB	15.8 ± 1.3aB	15.0 ± 1.0aB	14.7 ± 1.1aB	15.8 ± 1.3aB	12.3 ± 0.9bB
	Roots	-	-	-		22.2 ± 1.1aA	17.9 ± 1.0bA
TDS	Leaves	19.9 ± 1.2bA	25.1 ± 1.7aA	26.6 ± 1.7aA	24.5 ± 1.3aA	8.2 ± 0.7dB	13.7 ± 1.0cA
(mg·kg^-1^ d.w.)	Petioles	10.0 ± 0.8cB	12.8 ± 0.9bB	13.0 ± 1.0bB	11.9 ± 1.0bcB	6.9 ± 0.6cB	15.4 ± 1.1aA
	Roots	-	-	-		14.0 ± 1.0aA	14.6 ± 1.1aA
Monosaccharides	Leaves	3.9 ± 0.3bB	4.7 ± 0.4aB	4.7 ± 0.4aB	5.2 ± 0.4aB	4.4 ± 0.4abB	3.2 ± 0.3cB
(g·100 g^-1^ d.w.)	Petioles	7.5 ± 0.6bA	7.6 ± 0.6bA	9.7 ± 0.8aA	8.2 ± 0.7abA	7.1 ± 0.6bcA	6.3 ± 0.5cA
	Roots	-	-	-		7.5 ± 0.7aA	6.3 ± 0.6aA
Disaccharides	Leaves	2.3 ± 0.2aB	1.2 ± 0.1bB	0.7 ± 0.1cB	1.0 ± 0.2bA	1.2 ± 0.1bB	2.4 ± 0.1aC
(g·100 g^-1^ d.w.)	Petioles	2.9 ± 0.2bA	3.4 ± 0.3bA	1.5 ± 0.1cA	1.3 ± 0.2cA	0.3 ± 0.05dC	5.0 ± 0.4aB
	Roots					9.5 ± 0.8bA	16.6 ± 1.1aA
Nitrates	Leaves	1050 ± 97cA	1300 ± 105bA	1400 ± 115bA	1290 ± 92bA	728 ± 62dA	4109 ± 310aA
(mg·kg^-1^ d.w.)	Petioles	490 ± 36cB	650 ± 54bB	650 ± 52bB	704 ± 63bB	495 ± 35cB	4702 ± 342aA
	Roots	-	-	-		840 ± 71bA	1566 ± 123aB

The values followed by different letters are significantly different according to Duncan’s test at *p* ≤ 0.05; lowercase letters refer to the comparison between different genotypes and capital letters refer to the comparison between plant parts. Abbreviations: f.w., fresh weight; d.w., dry weight; TDS, total dissolved solids.

**Table 3:** Antioxidant status as affected by celery type and plant part.

		Celery Type					
Parameter	Plant Part	Leafy		Stalk		Root	
		Elixir	Samurai	Atlant	Primus	Egor	Dobrynya
Ascorbic acid (mg·100 g^-1^ f.w.)	Leaves	290 ± 25aA	229 ± 20bcA	254 ± 20abA	235 ± 16bcA	260 ± 20abA	216 ± 15cA
	Petioles	45.4 ± 1.6aB	47.7 ± 1.6aB	47.8 ± 1.0aB	46.0 ± 1.2aB	30.7 ± 2.5bB	28.1 ± 2.0bB
	Roots	-	-	-		30.9±2.7aB	33.3±2.8aB
Polyphenols (mg GAE.g^-1^ d.w.)	Leaves	17.2 ± 1.1aA	15.5 ± 1.0aA	16.0 ± 1.1aA	17.2 ± 1.0aA	15 ± 1.0abA	13.3 ± 0.9bA
	Petioles	10.0 ± 0.4aB	10.0 ± 0.4aB	8.6 ± 0.3bcB	9.0 ± 0.4bB	8.3 ± 0.3bcC	7.9 ± 0.3cC
	Roots	-	-	-		10.8 ± 0.4aB	10.8 ± 0.4aB
Flavonoids (mg-eq	Leaves	6.6 ± 0.2aA	4.8 ± 0.1bA	4.1 ± 0.1cA	3.8 ± 0.2cA	2.9 ± 0.1dA	3.7 ± 0.2cA
Q.g^-1^ d.w.)	Petioles	2.3 ± 0.1bB	3.0 ± 0.2aB	1.7 ± 0.1cB	1.5 ± 0.2cB	1.7 ± 0.1cB	2.4 ± 0.1bB
	Roots	-	-	-		1.2 ± 0.2aC	0.9 ± 0.1aC
AOA (mg GAE.g^-1^ d.w.)	Leaves	19.8 ± 1.3cA	28.4 ± 2.1bA	28.7 ± 2.1bA	28.0 ± 1.8bA	29.7 ± 2.2abA	33.6 ± 2.3aA
	Petioles	15.4 ± 1.0aB	12.3 ± 0.7bB	14.4 ± 0.9aB	14.0 ± 0.7aB	10.9 ± 0.4cC	11.9 ± 0.5bcC
	Roots	-	-	-		16.1 ± 1.1aB	16.3 ± 1.1aB

The values followed by different letters are significantly different according to Duncan’s test at *p* ≤ 0.05; lowercase letters refer to the comparison between different genotypes and capital letters refer to the comparison between plant parts. Abbreviations: f.w., fresh weight; d.w., dry weight; AOA, antioxidant activity; mg GAE, mg-equivalent of gallic acid; mg-eq Q, mg-equivalent of quercetin.

**Table 4:** Correlations between quality attributes, antioxidants and mineral elements in celery leaves and petioles.

	DM	NO_3_	TDS	AA	Fl	AOA	PP	MS	DS	Ash	K	Mn	Fe	Cu	Zn
NO_3_	−0.334	1													
TDS	0.422	0.053	1												
AA	0.918*	0.005	0.428	1											
Fl	0.701**	0.0393	0.608***	0.804	1										
AOA	0.756**	0.202	0.342	0.844*	0.484	1									
PP	0.943*	−0.106	0.542	0.978*	0.861*	0.781**	1								
MS	−0.676***	−0.381	−0.303	−0.848*	−0.718**	−0.791**	−0.801**	1							
DS	−0.606***	0.404	0.018	−0.451	-0.092	−0.524	−0.408	0.251	1						
Ash	0.391	0.584	0.380	0.554	0.260	0.782**	0.461	−0.631***	−0.292	1					
K	−0.163	−0.397	0.313	−0.276	0.095	−0.322	−0.121	0.448	0.244	−0.500	1				
Mn	0.366	0.379	−0.275	0.502	0.018	0.737**	0.353	−0.643***	−0.315	0.611***	−0.710**	1			
Fe	0.070	0.568	−0.323	0.253	−0.017	0.521	0.108	−0.600***	−0.326	0.515	−0.601***	0.774**	1		
Cu	0.096	0.730**	0.024	0.272	−0.020	0.515	0.146	−0.573	−0.067	0.821*	−0.794**	0.722**	0.693**	1	
Zn	0.570	−0.010	0.641***	0.667***	0.711**	0.625***	0.717**	−0.458	−0.174	0.336	0.437	0.049	−0.054	−0.166	1
Se	−0.029	0.095	−0.481	−0.111	−0.287	−0.001	−0.214	−0.126	−0.723	0.101	−0.676	0.320	0.594	0.471	−0.697**

***p < 0.001; **p < 0.01; *p < 0.05. Abbreviations: DM, dry matter; TDS, total dissolved solids; AA, ascorbic acid; Fl, flavonoids; AOA, antioxidant activity; PP, polyphenols; MS, monosaccharides; DS, disaccharides.

**Table 5:** Elemental composition as affected by celery type and plant part.

Parameter	Plant Part	Celery Type					
		Leaf		Stalk		Root	
		Elixir	Samurai	Atlant	Primus	Egor	Dobrynya
Ash (g·100 g^-1^ f.w.)	Leaves	9.6 ± 0.8cA	14.4 ± 1.5abA	17.2 ± 1.6aA	16.8 ± 1.5aA	13.0 ± 1.1bA	16.8 ± 1.4aA
	Petioles	7.9 ± 0.7cA	9.1 ± 0.8bcB	10.5 ± 1.0bB	10.3 ± 1.1bB	9.2 ± 0.9bcB	13.4 ± 1.2aB
	Roots	-	-	-		4.1 ± 0.3bC	6.0 ± 0.5aC
K	Leaves	36.2 ± 2.6aB	34.4 ± 2.0aB	28.3 ± 1.7bB	28.0 ± 1.4bB	15.5 ± 1.1cC	28.3 ± 1.1bA
(g·kg^-1^ d.w.)	Petioles	46.4 ± 3.5aA	48.0 ± 3.9aA	39.3 ± 2.5bA	38.6 ± 2.2bA	22.4 ± 1.8cA	20.7 ± 0.9cC
	Roots	-	-	-		18.6 ± 1.0bB	25.6 ± 1.2aB
Mn (mg·kg^-1^ d.w.)	Leaves	8.1 ± 0.7cA	13.1 ± 1.2bA	12.9 ± 1.2bA	13.2 ± 1.2bA	28.6 ± 2.9aA	24.9 ± 2.3aA
	Petioles	8.2 ± 0.9bA	6.1 ± 0.5cB	7.1 ± 0.6bcB	7.3 ± 0.8bcB	10.8 ± 1.0aB	13.3 ± 1.2aB
	Roots	-	-	-		8.5 ± 0.7aC	6.5 ± 0.7bC
Fe (mg·kg^-1^ d.w.)	Leaves	96.2 ± 8.5dA	125.8 ± 11.5cA	107.3 ± 9.9cdA	105.0 ± 10.1cdA	189.3 ± 17.5bA	342.5 ± 30.7aA
	Petioles	91.3 ± 8.8cA	53.8 ± 5.2dB	48.4 ± 4.3dB	46.7 ± 3.9dB	202.6 ± 19.0aA	151.3 ± 14.0bB
	Roots	-	-	-		47.8 ± 4.3aB	54.2 ± 5.1aC
Cu (mg·kg^-1^ d.w.)	Leaves	2.5 ± 0.2bA	4.6 ± 0.5aA	5.3 ± 0.5aA	5.5 ± 0.6aA	5.2 ± 0.5aA	5.7 ± 0.6aA
	Petioles	2.5 ± 0.3cA	2.6 ± 0.3cB	2.4 ± 0.2cB	2.5 ± 0.2cB	4.1 ± 0.3bB	6.2 ± 0.6aA
	Roots	-	-	-		4.1 ± 0.4bB	5.6 ± 0.6aA
Zn (mg·kg^-1^ d.w.)	Leaves	10.1 ± 0.9aA	8.9 ± 0.8aA	9.2 ± 0.8aA	8.9 ± 0.8aA	6.4 ± 0.7bA	9.9 ± 1.0a
	Petioles	7.9 ± 0.8aB	7.3 ± 0.6aA	7.5 ± 0.8aA	7.7 ± 0.7aA	2.6 ± 0.3cB	4.2 ± 0.3b
	Roots	-	-	-		7.2 ± 0.6bA	9.1 ± 0.8a
Se (µg·kg^-1^ d.w.)	Leaves	26 ± 2bA	43 ± 3aA	30 ± 3bA	28 ± 3bA	44 ± 3aA	42 ± 4aB
	Petioles	22 ± 2cA	25 ± 2bcA	28 ± 2cA	27 ± 2cA	38 ± 4bA	75 ± 6aA
	Roots					38 ± 3aA	24 ± 2bC

The values followed by different letters are significantly different according to Duncan’s test at *p* ≤ 0.05; lowercase letters refer to the comparison between different genotypes and capital letters refer to the comparison between plant parts. Abbreviations: f.w., fresh weight; d.w., dry weight.
